# Evaluating Antibiotic Resistance in Urban Rivers and Coral Reefs of Belize: Evidence for Hotspots and a Potential Screening Tool

**DOI:** 10.1029/2025GH001427

**Published:** 2026-02-14

**Authors:** Ileana A. Galdamez, Karina Jimenez, Marisol Cira, Katie Osborn, Mariam Ayad, Christine M. Lee, Kai Patel, Bryan A. Galdamez, Ashlyn Sloan, Alexis Shenkiryk, Taylor Cason, Nicole Auil Gomez, Myles Phillips, Samir Rosado, Andria Rosado, Deepak R. Mishra, Emil A. Cherrington, Robert Griffin, Jennifer A. Jay

**Affiliations:** ^1^ Department of Civil and Environmental Engineering University of California, Los Angeles Los Angeles CA USA; ^2^ Jet Propulsion Laboratory California Institute of Technology Pasadena CA USA; ^3^ Department of Ocean Sciences University of California, Santa Cruz Santa Cruz CA USA; ^4^ Wildlife Conservation Society Belize City Belize; ^5^ Coastal & Marine Data Centre Coastal Zone Management Authority & Institute Belize City Belize; ^6^ Department of Geography Center for Geospatial Research University of Georgia Athens GA USA; ^7^ Earth System Science Center University of Alabama in Huntsville Huntsville AL USA; ^8^ NASA SERVIR Science Coordination Office Marshall Space Flight Center Huntsville AL USA

## Abstract

Antibiotic resistance is a significant threat to global public health and can disproportionately affect low‐ and middle‐ income countries (LMICs). There is a lack of studies focusing on antibiotic resistance in coral reef regions and environmental reservoirs in Central America. This study followed modified World Health Organization (WHO)'s Global Tricycle Surveillance protocols for the environmental sector to address these gaps. Water samples were collected from key areas in the lower Belize River, including above and below the Belize City, an open fish market, and sewage lagoon outfall, and coral reefs. Water samples underwent qPCR analysis for a suite of antibiotic resistance gene classes (sul1, sul2, ermF, tetA, and blaSHV), intI1, and 16S rRNA. Additionally, a subset of samples were tested for extended‐spectrum β‐lactamase (ESBL) *E. coli* and underwent shotgun sequencing. Results show that antibiotic resistance genes (ARGs) were highest and most diverse near Belize City, particularly near the treatment lagoons and open fish market. The coral reef regions had lower levels of antibiotic resistance though not void of their presence. This study is an application of a modified Global Tricycle Surveillance protocol integrated with qPCR‐ and metagenomics‐based characterization of environmental antibiotic resistance in understudied areas. Notably, data from this study indicated that ESBL‐*E. coli* could potentially be used as a screening tool for environmental antibiotic resistance, as it was only present at sites that had the highest levels of ARGs.

## Plain Language Summary

This study looked at antibiotic resistance in water samples from the Belize River and coral reefs in Central America, areas that are not well studied for this issue. Antibiotic resistance is a growing global health problem, especially in low‐ and middle‐income countries. We followed a modified version of the World Health Organization's protocols to test for antibiotic resistance genes in the water. We found that the highest levels of antibiotic resistance were near Belize City, especially around sewage treatment lagoons and an open fish market. While coral reefs had lower levels of antibiotic resistance, some resistance was still present. The study also showed that a type of bacteria, extended‐spectrum β‐lactamase‐*E. coli*, could be useful for detecting high levels of antibiotic resistance in the environment. This research helps fill knowledge gaps about antibiotic resistance in under‐explored areas, particularly in environmental settings like coral reefs and rivers.

## Key Points

- Antibiotic resistance genes (ARGs) highest near urban areas in key hotspot areas, with lower levels detected in nearby coral reefs
- Extended‐spectrum β‐lactamase‐*E. coli* shows potential as a screening tool for environmental ARGs
- Coral reefs not void of ARGs, indicating influence of anthropogenic pollution

## 1 Introduction

Antibiotic resistance has been widely regarded as a global health crisis since the mid 1900s (Levy, 1998; Neu, 1992). In 2019, about 1.27 million deaths were attributed to antibiotic resistance globally (Murray et al., 2022). Antibiotic resistance arises due through misuse and overuse of antibiotics among humans and animals. Though antibiotic resistance genes (ARGs) have evolved and exist in natural and remote environments, anthropogenic stressors such as heavy metals and pharmaceuticals can co‐select for ARGs in bacteria. Aside from clinical settings, environment compartments, such as air, soil, and water, play an important role in the proliferation and dissemination of antibiotic resistance (Bombaywala et al., 2021). Sources of anthropogenic activities that impact the human resistome include livestock operations (He et al., 2020; Luiken et al., 2020; Yang et al., 2020), land application of manure to crop fields (Chee‐Sanford et al., 2009; Cira et al., 2021, p. 2; Zhao et al., 2017), composting and biosolid production (Barancheshme & Munir, 2018; Hung et al., 2022; Jacobs et al., 2019), non‐point discharges (Almakki et al., 2019; Baral et al., 2018; Garner et al., 2017), and effluent from wastewater treatment plants that have industrial, hospital, and residential influents (Czekalski et al., 2012; Davis et al., 2020; Eramo et al., 2019; Proia et al., 2018; Rodriguez‐Mozaz et al., 2015). Thus, the need to monitor and understand the intersection between environment and humans in terms of antibiotic resistance is warranted (Dalton et al., 2020; Keenum et al., 2022; Liguori et al., 2022; Nielsen et al., 2021).

Antibiotic resistance disproportionately affects low‐ and middle‐income countries (LMICs) (Domínguez et al., 2021; Farrar, 1985; Vlieghe et al., 2009). In fact, multidrug resistant organisms are the main cause of healthcare‐associated infections in Latin America (Domínguez et al., 2021) which may be attributed to high population density, poor living conditions, ineffective healthcare systems, and poor quality of antibiotics. Additionally, poor infrastructure leads to sanitation and water issues resulting in inaccessibility to potable water, illegal discharges, untreated wastewater, and poor solid waste disposal (Domínguez et al., 2021; Fuhrmeister et al., 2023; Husaini et al., 2020). Though behavioral interventions have been proven to have positive impact on use of antibiotics in LMICs (Cuevas et al., 2021), antibiotic resistance in these countries is still prevalent (Iskandar et al., 2021; Nadimpalli et al., 2018; Sulis et al., 2022). A review of studies conducted in Latin America found that plasmid‐mediated quinolone resistance genes are prevalent in humans, animals, food, and the environment (Vieira et al., 2020). Antibiotic resistant *Helicobacter pylori* had also been identified in Latin America and the Caribbean (Martínez et al., 2014; Ortiz et al., 2019). However, disparities among studies conducted within LMICs also exist. For example, most studies in Latin America are in Brazil and Mexico (Reichert et al., 2019) with not many investigations being conducted in Central America. There are very few studies that elucidate the impacts of antibiotic resistance in coral reefs, with all known studies being concentrated in China (Dong et al., 2022; Liu et al., 2020; Zhongjie et al., 2022; Zhou et al., 2024) and India (Hussain et al., 2023). The urgent need to monitor antibiotic resistance in other LMICs and various environmental matrices such as coral reefs and coastal areas still remains.

Belize, a country in Central America, has previously identified antibiotic‐resistant bacteria (ARB) as an issue in its healthcare system. Though antibiotics are prohibited for sale without a prescription through the Belize Antibiotic Act, a study showed that around 47.2% of surveyed community pharmacies were willing to sell antibiotics without a prescription most likely due to seeking of economic gain, weak regulatory enforcement, and consumer demands (Husaini et al., 2021). Another survey of Belizean college students reported that around 29% of students self‐medicate with antibiotics, which may potentially lead to adverse drug reactions, bacterial resistance, and drug interactions (Husaini et al., 2019). Children from San Pedro were found to have tetracycline‐ and sulfonamide‐resistant Enterobacteriaceae (Shears et al., 1988) and 56.97% of *Neisseria gonorrhoeae* isolates collected were penicillinase‐producing (Dillon et al., 2006). Belize has a large tourism economy accounting for over 40% of gross domestic product (GDP) (Cheng et al., 2021) and many international visitors chose to recreate along Belize's coastline, cayes, and atolls. There are several potential inputs of pollution into Belizean waterways such as agricultural runoff, waste, nutrients, and sewage (Emrich et al., 2017; Felgate et al., 2021; Lapointe et al., 2021; Silburn et al., 2023; Wells et al., 2019). Particularly, high tourism and poor sewage system is thought to be the reason for ARB in nurse sharks near the Hol Chan Marine Reserve (Blackburn et al., 2010). There are also anecdotes of community members experiencing itching, rashes, and other health effects from recreating in rivers (Drexler, 2020). As the nation continues to develop, the need to elucidate sources of ARGs and ARB in waterways is crucial to ensure the safety of Belizeans and tourists alike.

Current gaps in the AR surveillance in LMICs involve sampling the environmental resistome to understand resistance determinants and potentially contributing niches (Iskandar et al., 2021). There are multiple ways to monitor antibiotic resistance in waterways such as use of qPCR, sequencing and metagenomics, and culture‐based methods (Keenum et al., 2022; Miłobedzka et al., 2022; Nguyen et al., 2021). However, there is a need to cross‐validate between different AR monitoring methods and move toward greater application of standardized protocols (Davis et al., 2020; Keenum et al., 2022). The World Health Organization (WHO) has a set of standard protocols under the Global Tricycle Surveillance for extended‐spectrum β‐lactamase (ESBL)‐producing *E. coli* (ESBL‐EC) aimed at monitoring AR in humans, animals, and the environment, according to a One Health approach. The Tricycle approach deemed ESBL‐EC to be an appropriate proxy for monitoring AR because it exists across the three compartments under study, its abundance varies significantly between and within different countries, and it does account for some burden of disease. For environmental samples, a study conducted in a major city would consist of sampling from surface waters downstream and upstream from the city, near wet market or poultry market areas, and wastewater treatment plant inputs (World Health Organization, 2021). While wider application of the Tricycle protocol is desired, there are significant barriers to its use, including cost and availability of lab resources (Ruppé, 2023). The original protocol involves a labor intensive method for ESBL‐EC detection, while this study leverages recent work presenting a modified IDEXX‐based analysis that can quantify ESBL‐EC at significantly lower cost and with less labor (Hornsby et al., 2022; Jimenez et al., 2025). Additionally, Davis et al. describes an integrated approach to AR monitoring using qPCR and metagenomics analysis (Davis et al., 2020).

Integrating the Davis approach and WHO standard protocols, the purpose of this study was to assess ARGs and ARB using qPCR and metagenomics in the lower Belize River watershed, reef system near Belize City, and Glover's Reef Atoll. Five ARGs (sul1, sul2, ermF, tetA, and blaSHV) were quantified through qPCR, along with intI1 and 16S rRNA at 18 locations in Belize where a subset of six samples were sent for shotgun sequencing. Nutrients (nitrate, phosphate, total nitrogen, and ammonia), total organic carbon (TOC), and turbidity were also measured at all sites. To our knowledge, this study is the first of its kind in comprehensively surveying antibiotic resistance in Belize and Central America.

## 2 Materials and Methods

### 2.1 Study Area

Belize is a small Central American country bordered by Mexico, Guatemala, and the Caribbean Sea. Belize is in the heart of the Mesoamerican Barrier Reef System which is the world's second largest barrier reef. The Belize Barrier Reef Reserve System (BBRRS) is a UNESCO World Heritage Site which encompasses a variety of the country's biodiverse ecosystems including seagrass, coral reefs, and mangroves (UNESCO, 1996). The reef system contributes about 30% of the nation's GDP through coastal protection, fisheries, and tourism (Cho, 2005; Claudino‐Sales, 2019). The BBRRS has seven protected sites which include the Glover's Reef Marine Reserve established in 1993 (Cherrington et al., 2020). Glover's Reef Atoll (260 km^2^) is 45 km offshore from Belizean coastline and contains around 850 coral reef patches in its interior lagoon (Huntington et al., 2011). Glover's Reef has designated zones for spawning, general use, and conservation. Glover's Reef is home to a variety of fish species (Babcock et al., 2013; Flowers et al., 2022) and the Glover's Reef Research Station owned by the Wildlife Conservation Society is located in the reef's conservation zone.

The Belize River is the largest river in Belize running across the center of the country and flowing from the Peten District of Guatemala to the Belize coastal lagoon. The river outpours near Belize City, the most populous city in Belize (around 60,000 residents) and is approximately 290 km in length. It's a source for drinking water, tourism, fisheries, and mangrove cayes. Excessive nutrient loading, sediments, and pesticides have depleted the water quality of the river in part from deforestation and unsustainable agricultural practices. The pour point of the Belize River is located around 20 km away from the BBRRS. Studies have found trace metals, color dissolved organic matter, and nitrogen loading enrichment in the reef system from land‐based pollution (Felgate et al., 2021; Lapointe et al., 2021; Prouty et al., 2008). Belize Water Services provides wastewater treatment services in Belize City through a two‐cell facultative lagoon system with a 10‐day hydraulic retention time in each cell in series. The effluent is then discharged through a mangrove wetland into the Caribbean Sea (https://www.bws.bz/wastewater‐treatment.html).

### 2.2 Sample Collection and Filtration

**Figure 1:**
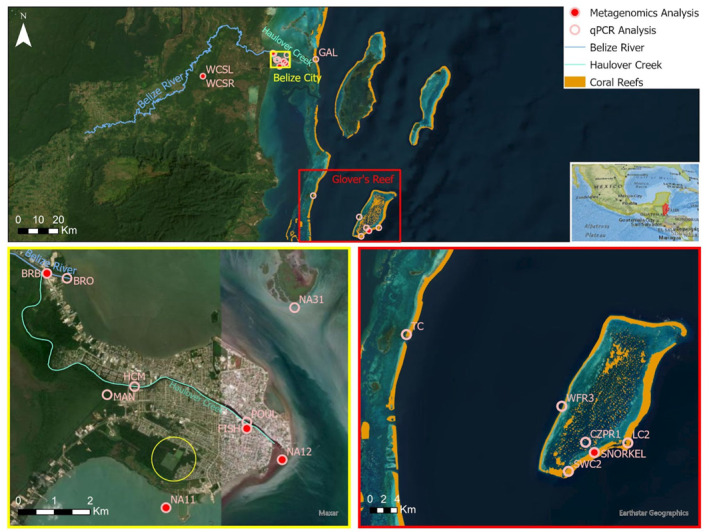
Sampling locations in the lower Belize River watershed and Belize coastal lagoon. The top panel shows all of the sampling locations (pink circles) between inland forested areas and coral reefs. The subset of samples sent for shotgun sequencing are filled in red. The bottom yellow panel depicts where the Belize River outpours to the coastal lagoon in the top left corner and Haulover Creek flows through Belize City. In this panel, the yellow circle in the bottom center is the wastewater treatment lagoon system. The bottom red panel shows the Glover's Reef and Tobacco Caye sampling locations.

Water samples were collected at 18 sites in Belize (Figure 1, Table 1): eight within the lower portion of the Belize River watershed, three near the surrounding coast of Belize City, two in coral areas in the BBRRS, and five locations at Glover's Reef Atoll. Water samples were taken from the surface (0–0.5 m depth) using sterile polypropylene bottles within a 4‐day time period between August and September 2022. Bottles were rinsed three times with ambient water before collection, transported on ice to the laboratory, and processed within 12 hr of collection.

**Table 1:** Name and Coordinates of Sampling Sites

Name	Date	Description	Latitude	Longitude
BRB	8/29/22	Belize River bridge	17.535921	−88.241917
BRO	8/29/22	Belize River outpour	17.534666	−88.236874
MAN	8/29/22	Mangrove canal	17.50556	−88.22691
HCM	8/29/22	Upper Haulover Creek	17.50763	−88.22006
POUL	8/29/22	Downstream from poultry operations	17.49872	−88.19202
FISH	8/29/22	Near fish market	17.49715	−88.19202
NA12	8/29/22	Haulover Creek outpour	17.48931	−88.18317
NA11	8/29/22	Near wastewater treatment lagoons	17.4773333	−88.21216667
NA31	8/30/22	Offshore Belize City	17.52735	−88.18008333
GAL	8/30/22	Gallow's Point Reef	17.50889	−88.05099
TC	8/31/22	Tobacco Caye	16.89898	−88.06194
WFR3	8/31/22	Glover's Reef: West/Forereef	16.80375	−87.85625
CZPR1	8/31/22	Glover's Reef: Conservation zone/Patch reefs	16.75611	−87.82483
LC2	8/31/22	Glover's Reef: Long Caye	16.75541	−87.76903
SNORKEL	8/31/22	Glover's Reef: Glover's Reef Research Station	16.74251	−87.8131
SWC2	8/31/22	Glover's Reef: Southwest Caye	16.71761	−87.8476
WCSL	9/1/22	Inland Belize River: Lagoon	17.4327187	−88.5546919
WCSR	9/1/22	Inland Belize River: River	17.4325622	−88.5545589

At the laboratory, water samples were filtered on 0.2 μm polycarbonate filters (MilliporeSigma, Burlington, MA). Volume of water necessary to clog the filter ranged from 100 to 750 mL and was recorded for each replicate. Filters were stored in 2 mL screw cap tubes with flame sterilized tweezers and fixed with 50% ethanol. Samples were stored at −20°C prior to arrival in the US. Filters were flown to the US on ice and stored at −20°C immediately upon arrival until DNA extraction. A trip blank was also taken and filtered.

### 2.3 DNA Extraction and qPCR

To prepare for the DNA extraction, filters were cut into approximately 1 cm^2^ pieces with flame‐sterilized scissors and placed into lysing matrix tubes from the FastDNA SPIN Kit for Soil (MP Biomedicals, Irvine, CA). The remaining ethanol solution was subjected to centrifugation at 5,000 × g for 10 min before being resuspended with the sodium phosphate buffer contained in the DNA kit and added to the lysing matrix tube. All samples were extracted according to manufacturer instructions taking the longest suggested times for incubation, mixing, and settling. An extraction blank was extracted per extraction batch. Total DNA concentrations and 260/280 absorbance ratios were determined through spectrophotometry via a NanoDrop 2000c (Thermo Scientific, Waltham, MA). Blank extracts yielded negligible nucleic acid concentrations and thus were not carried forward in the subsequent analysis.

Five ARGs, *sul*1, *sul*2, *erm*F, *te*tA, and *bla*SHV, were targeted for analysis through qPCR along with *intI*1 (a proxy for anthropogenic pollution) and the 16S rRNA gene (a surrogate for total bacteria). The selection of these ARGs was hypothesis‐driven to provide a representative snapshot of resistance mechanisms and antibiotic classes relevant to aquatic environments. Specifically, genes conferring resistance to common antibiotic classes were chosen: *sul*1 and *sul*2 (sulfonamides), *tet*A (tetracyclines), and *erm*F (macrolides). The inclusion of both *sul*1 and *sul*2 provides a more comprehensive assessment of sulfonamide resistance, as both are highly prevalent in aquatic environments. The *bla*SHV gene was selected to cover the extended‐spectrum β‐lactamase (ESBL) class and serve as a molecular complement to the culture‐based methods for ESBL‐producing *E. coli* used in this study. The *intI*1 gene was included as it is strongly associated with anthropogenic sources of contamination and often co‐locates with other ARGs, particularly *sul*1. Gene target sequences are in Table S1 in Supporting Information S1.

qPCR amplification was performed using the StepOnePlus system (Applied Biosystems, Foster City, CA) in 25 μL reaction volumes containing 12.5 μL PowerUp SYBR Green Master Mix (Applied Biosystems, Foster City, CA), 1.25 μL of each forward and reverse primer, 2 μL of template DNA, and molecular grade water for the remaining volume for all genes except 16S rRNA. The 16S rRNA gene was performed in a 20 μL reaction volume with 10 μL of PowerUp SYBR Green Master Mix, 1 μL of each forward and reverse primer, 3 μL of template DNA, and molecular grade water for the remaining volume. All assays were performed in 96‐well plates. At least a five‐point standard curve was run with each plate utilizing double‐stranded gBlock gene fragments resuspended according to manufacturer instructions (IDT, Coralville, IA) and quantified on the NanoDrop 2000c (Thermo Scientific, Waltham, MA). Ten‐fold dilutions were carried out for the 16S rRNA gene to detect the presence of PCR inhibitors. The lowest qPCR efficiency was 90.6% and the lowest *R* ^2^ value was 0.998. The limit of detection was set based on the lowest standard per assay. The qPCR data were analyzed using the StepOne Software v2.3. The Minimum Information for Publication of Quantitative Real‐Time PCR Experiments (MIQE) guidelines for the qPCR assays are located in Table S2 in Supporting Information S1.

### 2.4 Sequencing

Six locations were selected to send for sequencing which include upstream and downstream of the Belize River, coral reef area, and other key locations near the treatment lagoon outfall and open fish market. Approximately 100 ng of DNA from the subset were sequenced via paired‐end shotgun metagenomic sequencing (2 × 150 bp) on an Illumina NovaSeq 6000 by Mr. DNA (Shallowater, TX). Each library ranges from 10 to 30 million paired‐end sequences. All sequence data were deposited into the public NCBI Short Read Archive (SRA) database (#PRJNA1164810). The essential information for the Minimum Information about any (x) Sequence (MIxS) reporting standard for genomic data are in Tables S3 and S4 in Supporting Information S1 (Eloe‐Fadrosh et al., 2024). The raw sequences were uploaded into Galaxy (https://usegalaxy.org) for processing and assembly. Trimmomatic (Galaxy Version 0.38.0) was used to remove low quality reads from our pair‐end data (Bolger et al., 2014). The following Trimmomatic operations were used: SLIDINGWINDOW: 4, 20, MINLEN: 50, and AVGQUAL: 20. The paired data were assembled using de novo metagenomic assembly by MEGHIT (Galaxy Version 1.2.9) using the default settings of 2 for minimum multiplicity and 200 bp for the minimum length of output contigs (Li et al., 2015). FastQC (Galaxy Version 0.73) and Fasta Statistics (Galaxy Version 2.0) were used for quality control. Open reading frames on the contigs were predicted using Prodigal v2.6.3 (Hyatt et al., 2010).

ARGs were characterized using the ARGs‐OAP pipeline (v2.3) which uses the Structured Antibiotic Resistance Genes database to quantify ARG subtypes by cell number and 16S rRNA (Yin et al., 2018). Environmental Resistome risk (RR) scores were calculated using the MetaCompare pipeline (Oh et al., 2018). Assembled contigs were annotated using Prodial to predict protein‐coding genes (Hyatt et al., 2010). The sequences are then searched against the CARD database for ARGs, the ACLAME database for MGEs, and the PATRIC database for human pathogens. A risk score based on the co‐occurrence of ARGs, MGEs, and pathogens on assembled contigs in a 3D hazard space and sample coordinates are compared to a theoretical maximum risk point, with scores calculated as the inverse of this distance in log‐scale so that higher scores indicate greater RR.

Read‐based taxonomic classification was performed via the Centrifuge (v1.0.4) software on the National Microbiome Data Collaborative EDGE bioinformatics platform (https://nmdc‐edge.org/), which has previously been used for environmental metagenomic samples (Gweon et al., 2019).

### 2.5 Nutrients and Turbidity

Total organic carbon (TOC), nitrate, phosphate, total nitrogen, and ammonia concentrations were measured in mg/L for all samples using a Hach DR 2800 spectrophotometer and a DRB 200 reactor block (Hach Company, Loveland, CO), following the manufacturer's protocols. TOC was quantified using Hach Method 10129, which employs persulfate oxidation and UV digestion. Nitrate and total nitrogen were measured using the Hach TNTplus® 835 and 826 methods, respectively. Phosphate concentrations were determined using Hach Method 8190, based on the ascorbic acid reaction, while ammonia levels were measured using the Salicylate Method (Hach Method 8038). Sample preparation, digestion, and analysis followed Hach guidelines, with calibration and quality control steps performed before each measurement. All URLs for the test kits are in Table S5 in Supporting Information S1.

Turbidity was measured on an Orion AQUAfast AQ3010 turbidity meter (Thermo Scientific, Waltham, MA) in NTU.

### 2.6 Fecal Indicator Bacteria and Antibiotic Resistant *E. coli* Enumeration

Fecal indicator bacteria and ESBL‐EC were measured in areas samples taken in Belize City and surrounding coast (*n* = 8). FIB enumeration includes quantifying levels of total coliforms, *E. coli* (Colilert‐18, IDEXX), and Enterococci (Enterolert, IDEXX) bacteria using standard methods and kits (IDEXX Laboratories, Westbrook, ME). Final concentrations were reported in MPN/100 mL. Marine samples and samples along the main stem of the river were diluted 10‐fold and up to 1,000‐fold respectively according to manufacturer recommendations. For quantifying ESBL‐EC, 100 μL of 1 mg/mL cefotaxime was added to each prepared 100 mL IDEXX bottle with Colilert‐18 media. Samples were diluted at most 10‐fold.

### 2.7 Statistical Analysis

Data were analyzed and visualized in RStudio (v4.0.2) and Python (v3.11.8). Correlation plots were created with the “corrplot” (v0.90) package. ANOVA testing was performed in RStudio for the nutrients and turbidity data. The principal component analysis plot was created using the “prcomp” function and “ggbiplot” (v0.55) package. The “ggplot2” (v3.3.6) package was used to create all bar plots. The 3D plot of the RR factors was created using the “plot3D” (v1.4) package. Heatplots and chord plots were created using the “pheatmap” (v1.0.1) and “circlize” (v0.4.1) package, respectively. In Python, additional data processing was done with *pandas* (v1.5.3) to merge taxonomic data from multiple TSV files and calculate relative abundances at the genus and species levels. These calculations were visualized in a stacked bar plot using *matplotlib* (v3.6.0) to compare biodiversity across different samples. All data and scripts can be found on Github (https://github.com/iacallejas/Belize_ARG) and Zenodo (https://doi.org/10.5281/zenodo.15026578).

## 3 Results and Discussion

### 3.1 Concentrations of ARGs and intI1

**Figure 2:**
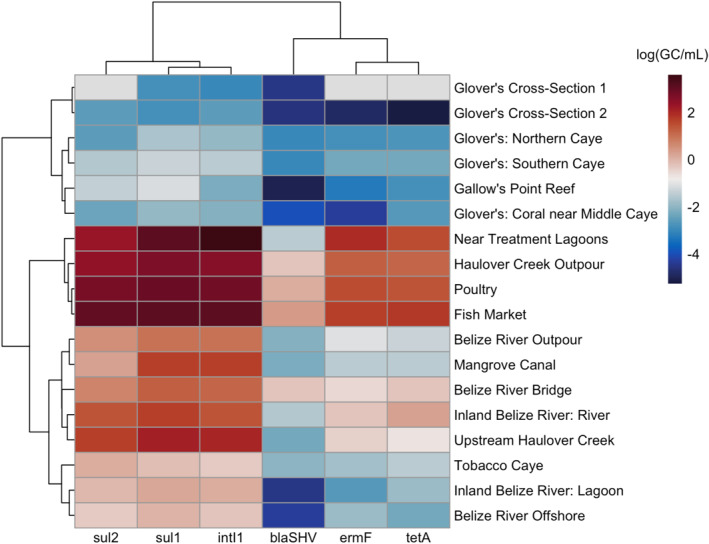
Absolute gene abundances for marine and river water samples with hierarchical clustering among genes and locations in gene copies per milliliter. Reef areas displayed the lowest absolute gene abundances while key hotspot sources near Belize City were highest.

In this study, five ARGs (*sul*1, *sul*2, *erm*F, *tet*A, and *bla*SHV) were quantified along with *intI*1 via qPCR. Figure 2 depicts the absolute abundances of ARGs and *intI*1 across 18 water samples. The genes *sul*1, *intI*1, and *sul*2 clustered together while *erm*F and *tet*A had similar trends. The sites displayed four general clusters with genes being the highest at through Haulover Creek in Belize City (FISH and POUL), the creek's pourpoint at the coast (NA12), and near the treatment lagoons discharge point (NA11). The next highest group encompasses the other inland samples from Belize River (Belize River Outpour, BRB, WCSR) and areas in Haulover Creek just before Belize City (MAN and HCM). The third group clustered together areas that are further from the coast of Belize (Tobacco Caye and NA31) and the other inland location in Belize River (WCSL). The sites with the lowest gene concentrations include all locations at Glover's Reef Atoll (WFR3, CZPR1, LC2, SNORKEL, SWC2) and the coral reef area closest to Belize City, Gallow's Point Reef (GAL).

Compared to a study in China that surveyed ARGs in water near corals, our ARG values (2.62 × 10^−3^ copies/L to 5.42 × 10^3^ copies/L) are significantly lower than theirs (7.34 × 10^4^ copies/L to 1.33 × 10^7^ copies/L) (Liu et al., 2020). The *sul*1 values in Belize are approximately three orders of magnitude lower than those in the South China Sea while the *sul*2 in our study are higher (Liu et al., 2020). This may be due to the larger populations on one of the islands studied which receive thousands of tourists per day. Another study in Chinese corals also had very high gene copies in their reefs (3.96 × 10^7^ to 1.65 × 10^9^ copies/L). In their case, *sul*2 was the most prevalent among their samples (Zhongjie et al., 2022).

### 3.2 AMR Trends via Metagenomic Data

**Figure 3:**
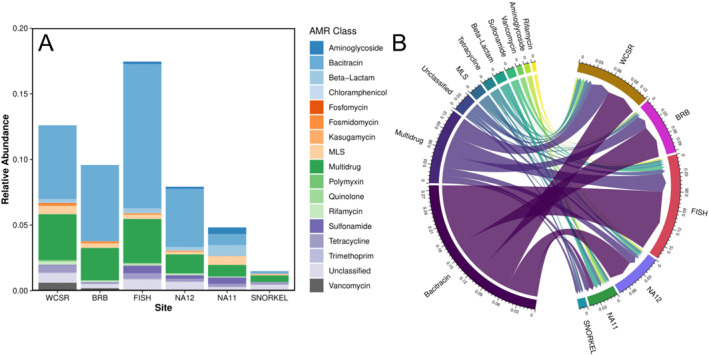
(a) Stacked bar plot 17 AMR classes and (b) chord plot of relative gene abundances of the top 10 AMR classes of the following locations: Inland Belize River (WCSR), Belize River Bridge (BRB), Fish Market (FISH), Haulover Creek Outpour (NA12), near wastewater treatment lagoon (NA11), and Glover's Reef (SNORKEL).

Out of the 24 antibiotic classes searched for by the ARGsOAP pipeline, 17 were represented among the samples. The inland locations (WCSR, BRB, FISH) had the highest relative abundances and distribution of AMR classes compared to the coastal waters (NA12 and NA11) and coral reef location (SNORKEL) (Figure 3a). The fish market location had the highest gene levels while the coral reef location had the lowest. Figure 3b shows the distribution of the top 10 AMR classes among the six samples sent for sequencing. The top 10 antibiotic classes are bacitracin, multidrug, unclassified, MLS, tetracycline, beta‐lactam, sulfonamide, vancomycin, aminoglycoside, and rifamycin. Bacitracin, the top antibiotic class identified, is widely used in animal husbandry and in ointments to prevent larval infestations in the skin which has been documented in Belize (Richards & Brieva, 2000; Xu et al., 2018). This data also shows the fish market having the highest partition of AMR gene occurrences. 161 out of the 1,244 genes were found across the six samples with *bac*A, *mdt*B, and CpxR being the three most prevalent.

A similar study that utilized shotgun sequencing in the coastal waters near the capital of Uruguay also found differing AMR classes between surface waters influenced by sewage and beaches not near sewage pipes (Fresia et al., 2019). A study in China monitoring the influence of wastewater on antibiotic resistance in surface waters also found bacitracin to be most prevalent among samples. This study attributes this to the inability of the treatment system to eradicate bacitracin resistance genes and its common use for topical therapy and growth promotion in animals (Tang et al., 2021). A study in Puerto Rico found higher relative ARG abundances in urbanized surface waters compared to rural and less impacted areas. The most dominant AMR class represented in their samples were multidrug and beta‐lactam ARGs revealed through metagenomic data (Davis et al., 2020).

### 3.3 Microbial Diversity

Figures S1 and S2 in Supporting Information S1 display the species and genus‐level taxonomic classifications based on the Centrifuge software. After comparing the top 20 taxonomies for each site, there were 57 unique species represented. The top species represented are *Bacteroides uniformis* (WCSR, BRB, FISH, NA12, NA11, SNORKEL), *Bacteroides cellulosilyticus* (WCSR, FISH, NA12, NA11, SNORKEL), *Bacteroides ovatus* (WCSR, BRB, FISH, NA12, SNORKEL), *Faecalibacterium prausnitzii* (WCSR, BRB, FISH, NA12, SNORKEL), *Polynucleobacter necessarius* (WCSR, BRB, FISH, NA12, NA11), *Rhodocyclaceae bacterium* ICHIAU1 (WCSR, BRB, FISH, NA12, NA11), *Limnohabitans* sp. 103DPR2 (WCSR, BRB, FISH, NA12), *Bifidobacterium pseudocatenulatum* (WCSR, BRB, NA12, SNORKEL). *Bacteroides*, *Faecalibacterium*, and *Bifidobacterium* are all gut‐associated bacteria which indicate fecal pollution (Ahmed et al., 2016; Benevides et al., 2017). *Polynucleobacter necessarius* and *Limnohabitans* sp. 103DPR2 are ubiquitous in freshwater habitats (Hahn et al., 2009; Kasalický et al., 2018).

### 3.4 Resistome Risk

**Figure 4:**
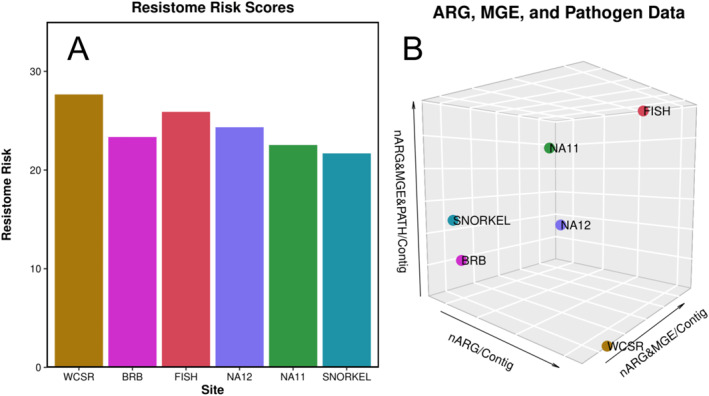
(a) Resistome risk scores and (b) 3D hazard space of co‐located antibiotic resistance gene, MGEs, and pathogens per contig using the MetaCompare pipeline of the following locations: Inland Belize River (WCSR), Belize River Bridge (BRB), Fish Market (FISH), Haulover Creek Outpour (NA12), Near wastewater treatment lagoon (NA11), and Glover's Reef (SNORKEL).

Resistome risk (RR) scores were calculated using the MetaCompare pipeline (Figure 4a). The inland most site (WCSR) had the highest RR score of 27.6 with the lowest, RR of 21.7, being the coral reef site at Glover's Reef Atoll (SNORKEL). Inland sites in both the Belize River (FISH) and Haulover Creek (BRB) were slightly higher than the corresponding pourpoint samples, NA12 and NA11. The three‐dimensional hazard space was plotted to visualize the co‐location of ARGs, MGEs, and pathogens as seen in Figure 4b. Here the fish market sample had the highest co‐location per contig of ARGs, MGEs, and pathogens while the inland Belize River site (WCSR) had the lowest.

Compared to RR scores calculated for a diversity of European water samples, the Belizean samples had scores resembling that on dairy lagoons and wastewater treatment plant effluent (Oh et al., 2018). WCSR, FISH, and NA12 sites had the highest RR scores among the samples (24.34–27.66) and were similar to the range of European dairy lagoons (22.71 < RR < 29.02). BRB, NA11, and SNORKEL had the lowest scores (21.69–23.35) and most closely resembled the RR scores of European wastewater treatment plant effluent (18.42 < RR < 22.77). Our values are also comparable to those found in water samples in Puerto Rico where rural areas had lower values (RR < 23) compared to surface waters in urban areas that have impacts of wastewater (RR > 25) (Davis et al., 2020). Surface water samples in coastal South Africa also had a RR score of 27.43 (Malla et al., 2025). In general, RR scores range from 18 to 28 in surface waters where waters with sewage influences may have similar or even lower scores than those that do not. The RR scores are better suited to distinguish among surface waters and sewage and wastewater treatment plant influent.

In terms of co‐location, FISH had the highest instances of contigs (*n* = 7) with the presence of ARGs, MGEs, and pathogens which resulted in a high RR score. Though WCSR did not exhibit any contigs with co‐location of ARGs with MGEs and pathogens, the higher ratio of ARGs per contig resulted in the highest RR score even compared to FISH which has the most co‐location. This shows that the RR score is greatly influenced by the hits against the CARD database and number of contigs. Other MetaCompare users have also calculated similar RR scores despite having variations among co‐located contigs, specifically for samples with decent water quality (Majeed et al., 2021).

### 3.5 Fecal Indicator Bacteria and ESBL‐*E. coli*

**Figure 5:**
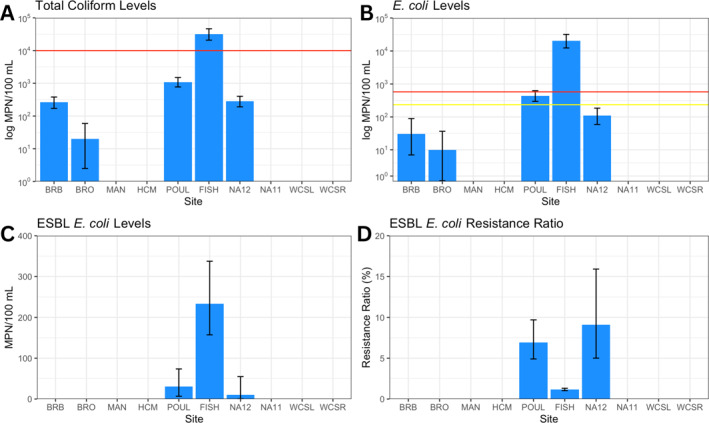
Results from IDEXX Colilert‐18 tests measuring total coliform (a) and *E. coli* levels (b). Graphs (c) and (d) are results from Colilert‐18 tests modified with Cefotaxime antibiotic. The red and yellow lines signify the USEPA standards for each respective bacteria type in recreational waters. The abbreviated location names are as follows: Belize River Bridge (BRB), Belize River Outpour (BRO), Mangrove canal (MAN), Upper Haulover Creek (HCM), Poultry operations (POUL), Fish market (FISH), Haulover Creek outpour (NA12), and near wastewater treatment lagoon (NA11).

Figures 5a and 5b depict levels of total coliform and *E. coli* measured by the IDEXX Colilert‐18 tests. From the eight sites measured, FIB levels were highest near the poultry (POUL) and fish market (FISH) along Haulover Creek. Total coliform and *E. coli* levels for FISH exceeded EPA standards, with POUL also being above recommendations for *E. coli*. With the addition of Cefotaxime to the Colilert‐18 tests (Figures 5c and 5d), ESBL‐producing *E. coli* were detected mainly at FISH, although the resistance ratio was highest for POUL and NA12.

### 3.6 Correlations Among ARGs, Nutrients, and FIB

Figure S3 in Supporting Information S1 shows the correlation coefficients for normalized ARGs, 16S rRNA, *intI*1, FIB, and nutrients. There were very strong positive correlations (*p* < 0.001) among all ARGs except *erm*F and *bla*SHV. The correlations between *sul*1, *sul*2, and *intI*1 are typically strong compared to those among macrolide and tetracycline resistance genes (Sabri et al., 2020; Wang et al., 2021). There were also strong positive correlations among FIB (*p* < 0.001) and FIB and ammonia (*p* < 0.05). Ammonia also had strong correlations with 16S rRNA (*p* < 0.001) and total nitrogen correlated with the *E. coli* resistance ratio (*p* < 0.05). The *bla*SHV gene also correlated with phosphate (*p* < 0.05) and ammonia (*p* < 0.05). A study in surface waters in Malaysia also found strong positive correlations among ARGs, ESBL *E. coli*, MGEs, and nutrients (Ott et al., 2021).

Figure S4 in Supporting Information S1 depicts boxplots of the nutrients and turbidity across the varying land development types (inland undeveloped, inland developed, coastal nearshore, coastal offshore, and Glover's Reef). Broad chemical nutrient tests for nitrate, phosphate, total nitrogen, and total organic carbon did not indicate any statistically significant differences between site types through ANOVA testing. Only turbidity and ammonia showed statistically significant differences between inland and reef sites. The inland undeveloped sites were significantly higher than Glover's Reef (*p* = 0.048) in turbidity, and inland developed sites were higher in ammonia compared to Glover's Reef (*p* = 0.028). These findings are corroborated by other studies in Belize, one finding that total suspended sediments are resuspended in coastal Belize due to boat traffic and other coastal activities (Maniyar et al., 2023). Increased nutrients such as nitrogen were also found to impact increased macroalgal blooms near the Belize Barrier Reef with Haulover Creek and the Belize River as a source (Lapointe et al., 2021). The excessive discharge of reactive nitrogen such as ammonia may indicate improper wastewater treatment which has recently been studied in Laughing Bird Caye in Belize (Delgado et al., 2025). These results indicate nuances of land‐based pollution to the reef. However, the lack of continuous monitoring is a limitation in our study.

**Figure 6:**
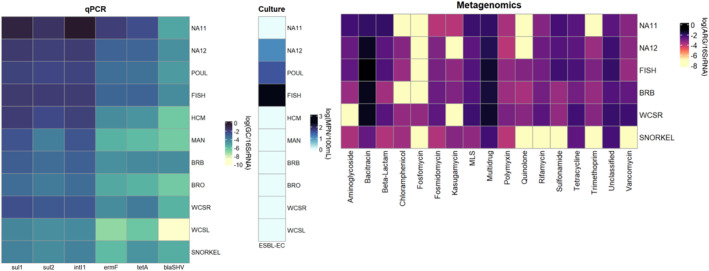
Heatplots of qPCR‐, culture‐, and metagenomic‐based antibiotic resistance determinants. The following locations are for qPCR analysis with subsets for culture‐based and metagenomic analysis: near wastewater treatment lagoons (NA11), Haulover Creek outpour (NA12), poultry operations (POUL), fish market (FISH), upper Haulover Creek (HCM), mangrove canal (MAN), Belize River Bridge, Belize River Outpour, inland Belize River (WCSR), inland Belize River lagoon (WCSL), and Glover's Reef (SNORKEL).

Notably, IDEXX‐based ESBL‐*E. coli* quantification served as a suitable indicator of the highest levels of ARGs detected by qPCR (at FISH, POUL, and NA12) as shown in Figure 6, with the exception being when the likely sources had been disinfected (NA11). This is an important result, as the WHO has deemed ESBL‐*E. coli* as an ideal target organism for monitoring antimicrobial resistance worldwide. Indeed, the WHO's Tricycle Protocol uses a One Health approach to characterize this organism in humans, non‐human animals, and the environment. While wider and repeated application of this standardized method would allow tracking of spatial and temporal trends in global AMR, so far only 19 countries have conducted the Tricycle Protocol. Further, only four have repeated the analysis, which is not surprising due to the significant implementation challenges (Ruppé, 2023). While repeated sampling would have allowed a more accurate characterization of the environmental conditions, this was not possible in this case due to the international travel and time required from local partners. The cross method comparison aspect of this study is valid for the snapshot design.

Challenges to more widespread application of the Tricycle Protocol including complexity of laboratory procedures, cost of the traditional methods for ESBL‐EC detection, and portability of the necessary laboratory equipment. The IDEXX‐based method addresses all of these issues. For this work, piloted an application of the environmental sampling portion of the Tricycle Protocol where airplane travel with almost all necessary lab equipment was required. While it was necessary to transport the IDEXX sealer and UV box, the rest of the needed equipment (bottles and incubator) was present at the field site. If needed, it would be possible to construct field‐portable incubators. Once at the field site, we did not have to seek out a Biosafety Level 2 lab; rather, with the IDEXX‐kits, the cultures are contained and thus, only Biosafety Level‐1 status is required. A requirement for Biosafety Level 2 status would reduce the applicability of the method. Reduced cost and user‐friendliness are additional advantages of the IDEXX‐based method.

## 4 Conclusions

In this study, various methods to monitor antibiotic resistance were conducted in a variety of aquatic regions in Belize, including coral reefs. This study integrated a modification of the WHO's Tricycle environmental surveillance framework, which calls for a collection of water samples in hotspot sources such as wet markets, wastewater discharge, and upstream and downstream of major cities, with snapshot qPCR‐ and metagenomics‐based approaches to characterize the resistome. The areas closest to Belize City, the most populated city in Belize, had elevated ARGs, particularly the fish market in Haulover Creek and the area of the coast closest to the treatment lagoon. These two areas also had the greatest instances of co‐location of ARGs, MGEs, and pathogens as revealed by metagenomic data and high FIB and ESBL *E. coli* by culture methods. Though coral reef areas had lower ARGs than the inland and coastal sites, they were not completely devoid of ARGs, indicating the influence of anthropogenic pollution. Notably, ESBL‐EC was only present at sites that had the highest levels of ARGs, indicating potential usefulness of this organism as a screening tool. In fact, the only sites with high ARG levels that didn't also show presence of ESBL‐EC was a coastal site near the outlet of the sewage treatment plant, where the effluent was presumably disinfected and therefore not amenable to culture‐based testing. Here, viability, amplification, and sequencing methods for monitoring antibiotic resistance proved to be useful in identifying these dynamic pollutants in an understudied part of the world. Thus, more studies should be conducted in Central America to reveal the threats of antibiotic resistance on human health and ecological impacts.

## Conflict of Interest

The authors declare no conflicts of interest relevant to this study.

## Supporting information

Supporting Information S1

## Data Availability

The data used for analyzing and visualizing ARGs, taxonomic diversity, and related resistome risk factors across water samples in the study are available at Github (https://github.com/iacallejas/Belize_ARG) and Zenodo (Callejas, 2025). All sequence data were deposited into the public NCBI Short Read Archive (SRA) database (#PRJNA1164810). These materials are open‐source and distributed under the terms of the MIT License.
