# Supplementary material for: Evaluating Antibiotic Resistance in Urban Rivers and Coral Reefs of Belize: Evidence for Hotspots and a Potential Screening Tool
Source: Geohealth. 2026 Feb 14;10(2):e2025GH001427. doi: 10.1029/2025GH001427 (PMC12905505; doi:10.1029/2025GH001427)
Supplement: Supplementary file 1 — Supporting Information S1 [file GH2-10-e2025GH001427-s001.docx]

**Supporting Information for:**

**Evaluating Antibiotic Resistance in Urban Rivers and Coral Reefs of Belize: Evidence for Hotspots and a Potential Screening Tool**

**Ileana A. Galdamez, Karina Jimenez, Marisol Cira, Katie Osborn, Mariam Ayad, Christine M. Lee, Kai Patel, Bryan A. Galdamez, Ashlyn Sloan, Alexis Shenkiryk, Taylor Cason, Nicole Auil Gomez, Myles Phillips, Emil A. Cherrington, Robert Griffin, Samir Rosado, Andria Rosado, Deepak R. Mishra, & Jennifer A. Jay**

*Corresponding author: Ileana Galdamez; [iacallejas@g.ucla.edu](mailto:iacallejas@g.ucla.edu)

**Number of Pages: 12**

**Number of Figures: 4**

**Number of Tables:** 5

**Table of Contents**

Figures: S1 – S4

Tables: S1– S5


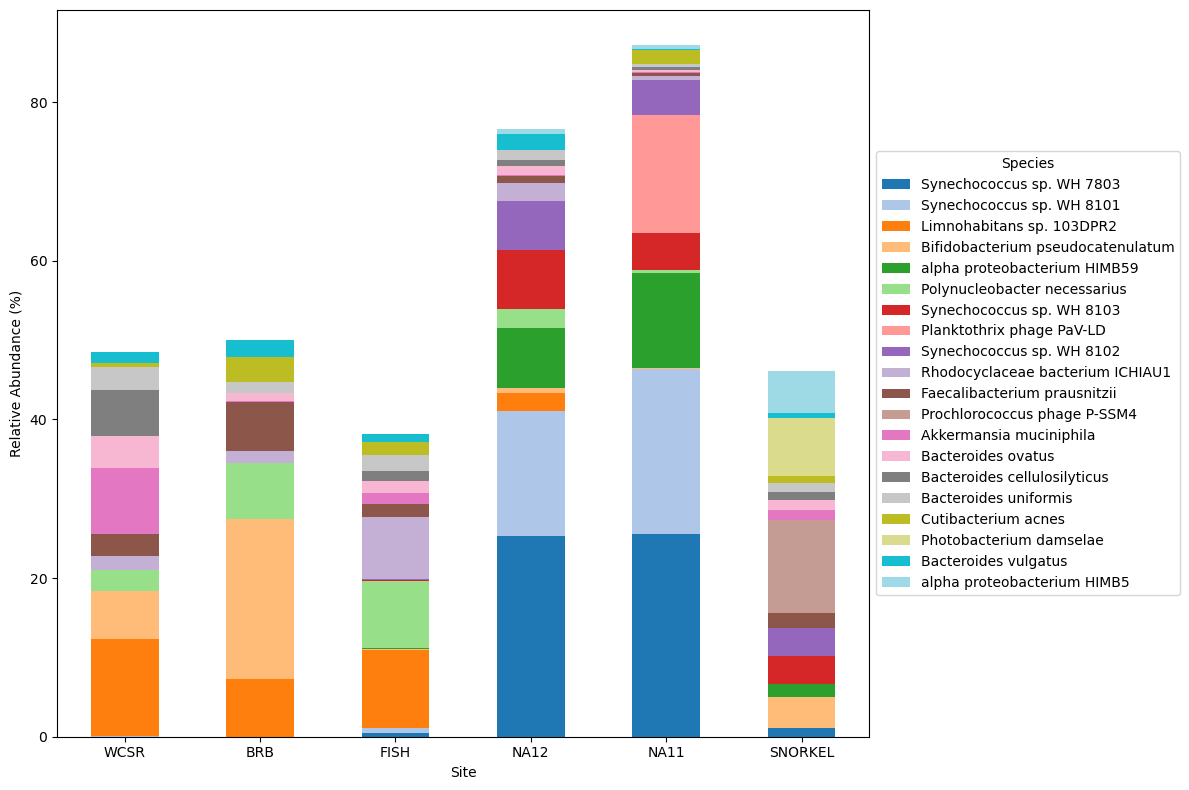


**Figure S1**. Top 20 species-level classifications identified through Centrifuge pipeline.


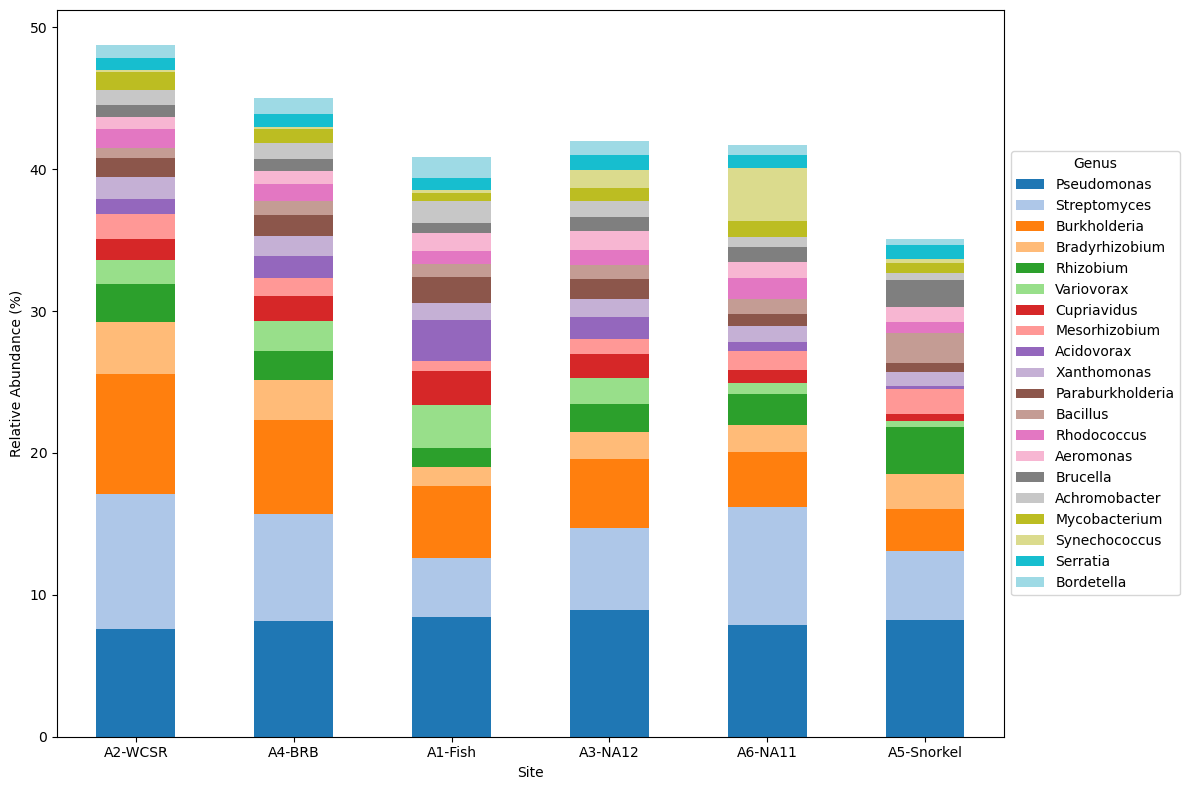


**Figure S2**. Top 20 genus-level classifications among sample subset through Centrifuge pipeline.

**
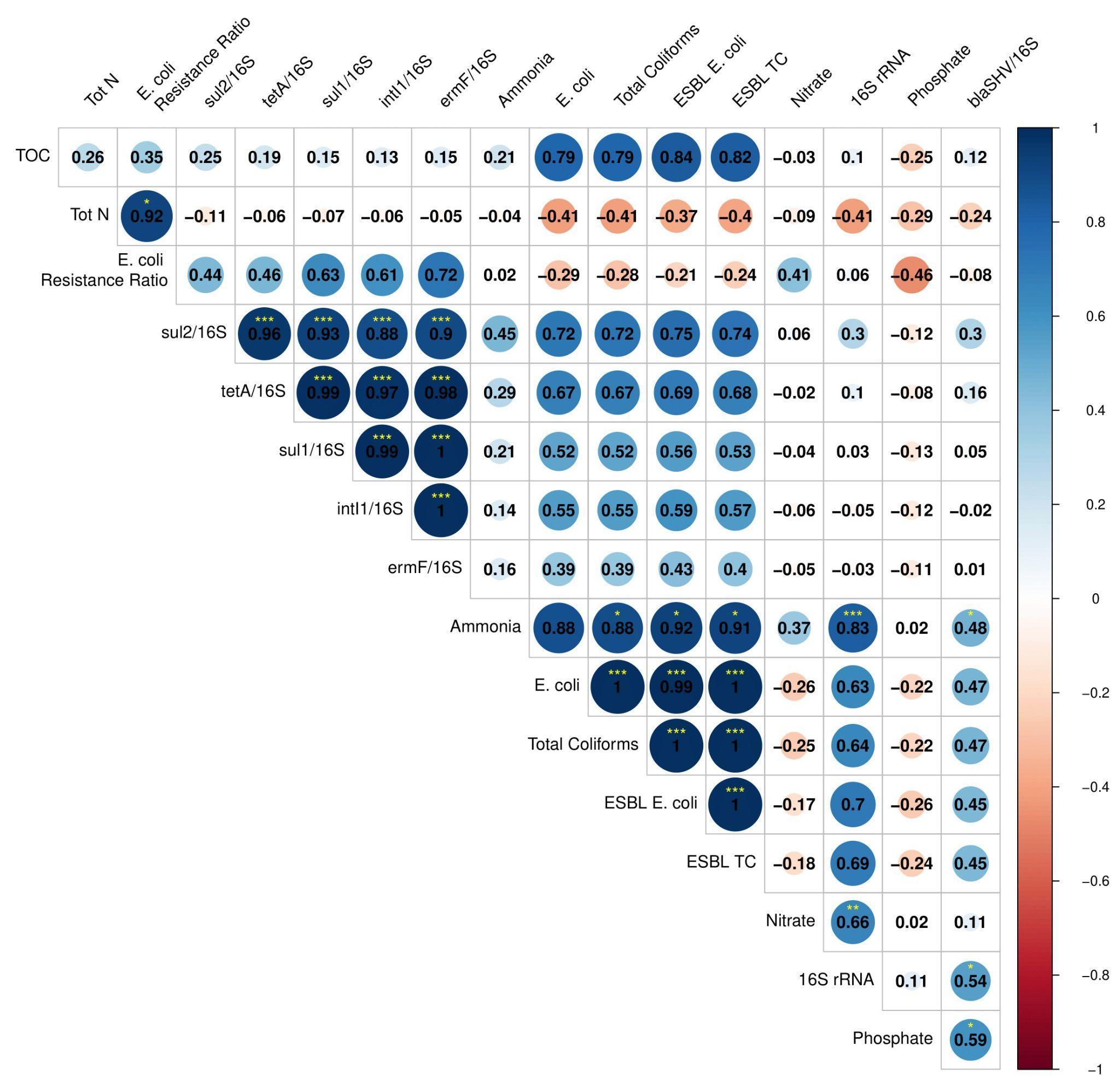
**

**Figure S3**. Correlation plot of ARGs, nutrients, and fecal indicator bacteria. One star ('*'), Two stars ('**'), Three stars ('***') denote p-values less than 0.05, 0.01, and 0.001, respectively.


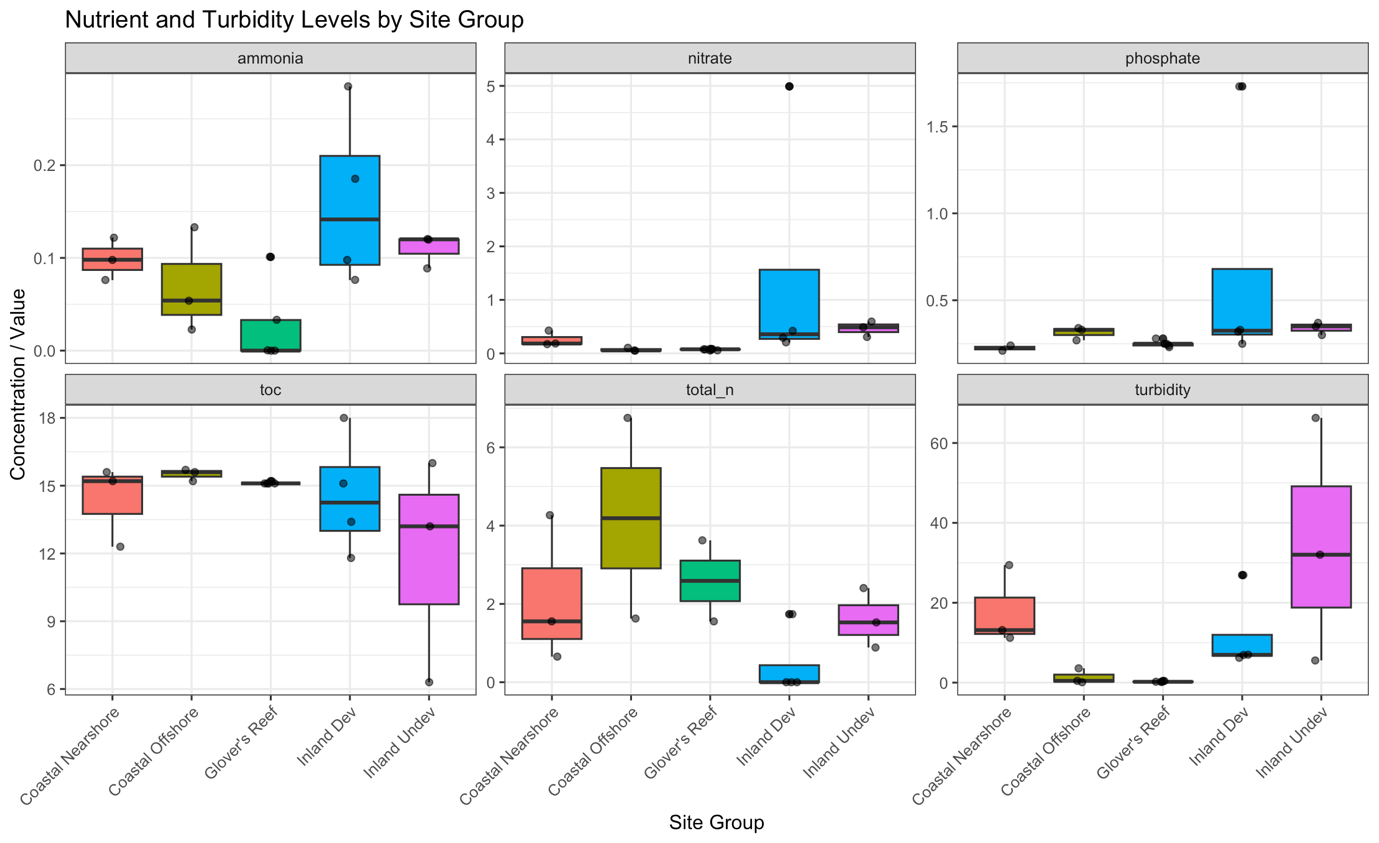


**Figure S4**. Boxplot of nutrients and turbidity by site category: Inland undeveloped (MAN, WCSL, WCSR), inland developed (BRB, FISH, HCM, POUL), coastal nearshore (BRO, NA11, NA12), coastal offshore (GAL, NA31, TC), and Glover’s Reef (CZPR1, LC2, SNORKEL, SWC2, WFR3).

**Table S1**. qPCR assay information.

| **Target** | | **Oligo** | **Size (bp)** | **Annealing Temperature (°C)** | **N of cycles** | **LOD** | **Efficiency (%)** | **R^2^** | **Reference** |
| --- | --- | --- | --- | --- | --- | --- | --- | --- | --- |
| **Gene** | **Name** | **Sequence (5'-3')** |  |  |  |  |  |  |  |
| sul1 | sul1-FW | CGCACCGGAAACATCGCTGCAC | 258 | 65 | 45 | 2.87 | 91.8535 | 0.9995 | (Pei et al., 2006) |
|  | sul1-RV | TGAAGTTCCGCCGCAAGGCTCG |  |  |  |  |  |  |  |
| intI1 | intI1-FW | GGCTTCGTGATGCCTGCTT | 424 | 55 | 45 | 1.5 | 95.4015 | 0.999 | (Luo et al., 2010) |
|  | intl1-RV | CATTCCTGGCCGTGGTTCT |  |  |  |  |  |  |  |
| ermF | ermF-FW | TCGTTTTACGGGTCAGCACTT | 246 | 50 | 45 | 2.59 | 97.7745 | 0.9995 | (Knapp et al., 2010) |
|  | ermF-RV | CAACCAAAGCTGTGTCGTTT |  |  |  |  |  |  |  |
| blaSHV | blaSHV-FW | TGATTTATCTGCGGGATACG | 215 | 55 | 40 | 3.99 | 89.6275 | 0.9995 | (Knapp et al., 2010) |
|  | blaSHV-RV | TTAGCGTTGCCAGTGCTCG |  |  |  |  |  |  |  |
| tetA | tetA-FW | GCTACATCCTGCTTGCCTTC | 250 | 55 | 45 | 4.11 | 95.559 | 1 | (Ng et al., 2001) |
|  | tetA-RV | CATAGATCGCCGTGAAGAGG |  |  |  |  |  |  |  |
| sul2 | sul2-FW | CTCCGATGGAGGCCGGTAT | 190 | 60 | 45 | 2.08 | 92.6135 | 1 | (Luo et al., 2010) |
|  | sul2-RV | GGGAATGCCATCTGCCTTGA |  |  |  |  |  |  |  |
| 16S rRNA | 16S-FW | CGGTGAATACGTTCYCGG | 124 | 56 | 40 | 16.9 | 91.7358 | 0.9994 | (Suzuki et al., 2000) |
|  | 16S-RV | GGWTACCTTGTTACGACTT |  |  |  |  |  |  |  |

*: FW, forward; RV, reverse

**Table S2**. Details of essential and desired information required in MIQE guidelines for antibiotic resistance gene detection (qPCR).

| **Item to check** | **Provided (Y/N)** | **Comment** |
| --- | --- | --- |
| **1. Sample** | | |
| Description | Y | Included in methods section |
| Volume/mass of sample processed | Y | Included in methods section |
| Processing procedure | Y | Included in methods section |
| Sample storage conditions and duration | Y | Included in methods section |
| **2. Nucleic acid extraction** | | |
| Procedure and/or instrumentation | Y | Included in methods section |
| Name of kit and details of any modifications | Y | Included in methods section |
| Details of DNase or RNase treatment | N | N/A |
| Contamination assessment (DNA or RNA) | Y | Included in methods section |
| Nucleic acid quantification | Y | Included in methods section |
| Instrument and method | Y | Included in methods section |
| Inhibition testing (Cq dilutions, spike, or other) | Y | Included in methods section |
| **3. qPCR target information** | | |
| Sequence accession number or official gene symbol | Y | Included in methods section |
| Amplicon length | Y | Included in SI |
| In silico specificity screen (BLAST, and so on) | N | Not done |
| Location of each primer by exon or intron (if applicable) | N | Not applicable |
| What splice variants are targeted? | N | Not done |
| **4. qPCR oligonucleotides** | | |
| Primer sequences | Y | Included in SI |
| Probe sequences | N | Not applicable |
| Location and identity of any modifications | N | Not done |
| **5. qPCR protocol** | | |
| Complete reaction conditions | Y | Included in methods section |
| Reaction volume and amount of DNA | Y | Included in methods section |
| Primer (probe) concentrations | Y | Included in methods section |
| Mg2+ and dNTP concentrations | N | Used commercial master mix |
| Polymerase identity and concentration | N | Used commercial master mix |
| Buffer/kit identity and manufacturer | Y | Included in methods section |
| Additives (SYBR Green I, DMSO, and so forth) | N | Not applicable |
| Complete thermocycling parameters | Y | Included in SI |
| Manufacturer of qPCR instrument | Y | Included in methods section |
| **6. qPCR validation** | | |
| Specificity (gel, sequence, melt, or digest) | N | Not done |
| For SYBR Green I, Cq of the NTC | N | Not done |
| Calibration curves with slope and y intercept | N | Not done |
| PCR efficiency calculated from slope | Y | Included in SI |
| r2 of calibration curve | Y | Included in SI |
| Linear dynamic range | N | Not done |
| Cq variation at LOD | N | Not done |
| Evidence for LOD | Y | Included in SI |
| **7. Data analysis** | | |
| qPCR analysis program (source, version) | Y | Included in methods section |
| Method of Cq determination | Y | Included in methods section |
| Outlier identification and disposition | N | Too few replicates to define outlier |
| Results for NTCs | N | Not included |
| Justification of number and choice of reference genes | N | Not applicable |
| Description of normalization method | N | Not done |
| Number and stage (reverse transcription or qPCR) of technical replicates | Y | Included in methods section |
| Repeatability (intraassay variation) | N | Not included |
| Statistical methods for results significance | Y | Included in methods section |
| Software (source, version) | Y | Included in methods section |
| Data transparency | Y | Data available by request |

**Table S3**. Details of essential information according to the Minimum Information about any (x) Sequence (MIxS) - MIMS (Metagenome or Environmental) checklist.

| **sample_name** | **env_local_scale** | **env_medium** | **samp_taxon_id** | **geo_loc_name** | **collection_date** | **seq_meth** | **lat_lon** | **env_broad_scale** |
| --- | --- | --- | --- | --- | --- | --- | --- | --- |
| A1_FISH_R1 | Urban river near fish market | Water | 1775375 | Belize | 8/29/22 | NovaSeq 6000 | 17.49715 N, 88.19202 W | Urban river |
| A1_FISH_R2 | Urban river near fish market | Water | 1775375 | Belize | 8/29/22 | NovaSeq 6000 | 17.49715 N, 88.19202 W | Urban river |
| A2_WCSR_R1 | Inland freshwater lagoon | Water | 1762686 | Belize | 9/1/22 | NovaSeq 6000 | 17.4325622 N, 88.5545589 W | Inland lagoon |
| A2_WCSR_R2 | Inland freshwater lagoon | Water | 1762686 | Belize | 9/1/22 | NovaSeq 6000 | 17.4325622 N, 88.5545589 W | Inland lagoon |
| A3_NA12_R1 | Coastal water | Seawater | 1239794 | Belize | 8/29/22 | NovaSeq 6000 | 17.48931 N, 88.18317 W | Marine environment |
| A3_NA12_R2 | Coastal water | Seawater | 1239794 | Belize | 8/29/22 | NovaSeq 6000 | 17.48931 N, 88.18317 W | Marine environment |
| A4_BRB_R1 | Urban river | Water | 1775375 | Belize | 8/29/22 | NovaSeq 6000 | 17.535921 N, 88.241917 W | Urban river |
| A4_BRB_R2 | Urban river | Water | 1775375 | Belize | 8/29/22 | NovaSeq 6000 | 17.535921 N, 88.241917 W | Urban river |
| A5_SNORKEL_R1 | Coral reef | Seawater | 1341441 | Belize | 8/31/22 | NovaSeq 6000 | 16.74251 N, 87.8131 W | Coral reef |
| A5_SNORKEL_R2 | Coral reef | Seawater | 1341441 | Belize | 8/31/22 | NovaSeq 6000 | 16.74251 N, 87.8131 W | Coral reef |
| A6_NA11_R1 | Coastal water | Seawater | 1239794 | Belize | 8/29/22 | NovaSeq 6000 | 17.4773333 N, 88.21216667 W | Marine environment |
| A6_NA11_R2 | Coastal water | Seawater | 1239794 | Belize | 8/29/22 | NovaSeq 6000 | 17.4773333 N, 88.21216667 W | Marine environment |

**Table S4.** DNA, final library concentration, and average library size.

The initial concentration of DNA (Table S4) was evaluated using the Qubit® dsDNA HS Assay Kit (Life Technologies). 50 ng DNA was used to prepare the library using Illumina DNA Prep, (M) Tagmentation library preparation kit (Illumina) following the manufacturer's user guide. The samples underwent the simultaneous fragmentation and addition of adapter sequences. These adapters are utilized during a limited-cycle PCR in which unique indices were added to the sample. Following the library preparation, the final concentration of the libraries (Table S4) was measured using the Qubit® dsDNA HS Assay Kit (Life Technologies), and the average library size (Table S4) was determined using the Agilent 2100 Bioanalyzer (Agilent Technologies). The libraries were then pooled in equimolar ratios of 0.6nM, and sequenced paired end for 300 cycles using the NovaSeq 6000 system (Illumina).

| **Sample ID** | **DNA concentration (ng/µL)** | **Final library DNA concentration (ng/µL)** | **Average Library**  **size (bp)** |
| --- | --- | --- | --- |
| A1_FISH | 42.6 | 27.6 | 775 |
| A2_WCSR | 6.9 | 40.8 | 641 |
| A3_NA12 | 21.4 | 37.6 | 649 |
| A4_BRB_R1 | 15.7 | 14.1 | 854 |
| A5_SNORKEL | 2.5 | 40 | 694 |
| A6_NA11 | 33.2 | 35.8 | 654 |

**Table S5.** Hach Company links and references.

| Test Name | URL | Reference |
| --- | --- | --- |
| Total Organic Carbon (TOC) Reagent Set, LR | https://www.hach.com/p-toc-tnt-vial-test/2760345 | (Hach Company, n.d.) |
| Nitrate TNTplus Vial Test, LR (0.23-13.5 mg/L NO₃-N) | https://www.hach.com/p-nitrate-tntplus-vial-tests/TNT835 | (Hach Company, n.d.) |
| Nitrogen (Total) TNTplus Vial Test, LR (1-16 mg/L N) | https://www.hach.com/p-total-nitrogen-s-tkn-tntplus-vial-tests/TNT826 | (Hach Company, n.d.) |
| Phosphorus (Total) TNT Reagent Set, Low Range | https://www.hach.com/p-phosphate-tnt-vial-test/2742645 | (Hach Company, n.d.) |
| Nitrogen-Ammonia Reagent Set, Nessler | https://www.hach.com/p-nitrogen-ammonia-reagent-set-nessler/2458200 | (Hach Company, n.d.) |

References

Hach Company, n.d. Total Organic Carbon (TOC) Reagent Set, LR [WWW Document]. URL https://www.hach.com/p-toc-tnt-vial-test/2760345 (accessed 8.11.25a).

Hach Company, n.d. Nitrate TNTplus Vial Test, LR (0.23-13.5 mg/L NO₃-N), 25 Tests [WWW Document]. URL https://www.hach.com/p-nitrate-tntplus-vial-tests/TNT835 (accessed 8.11.25b).

Hach Company, n.d. Nitrogen (Total) TNTplus Vial Test, LR (1-16 mg/L N), 25 Tests [WWW Document]. URL https://www.hach.com/p-total-nitrogen-s-tkn-tntplus-vial-tests/TNT826 (accessed 8.11.25c).

Hach Company, n.d. Phosphorus (Total) TNT Reagent Set, Low Range [WWW Document]. URL https://www.hach.com/p-phosphate-tnt-vial-test/2742645 (accessed 8.11.25d).

Hach Company, n.d. Nitrogen-Ammonia Reagent Set, Nessler [WWW Document]. URL https://www.hach.com/p-nitrogen-ammonia-reagent-set-nessler/2458200 (accessed 8.11.25e).

Knapp, C.W., Dolfing, J., Ehlert, P.A.I., Graham, D.W., 2010. Evidence of Increasing Antibiotic Resistance Gene Abundances in Archived Soils since 1940. Environmental Science & Technology 44, 580–587. https://doi.org/10.1021/es901221x

Luo, Y., Mao, D., Rysz, M., Zhou, Q., Zhang, H., Xu, L., J. J. Alvarez, P., 2010. Trends in Antibiotic Resistance Genes Occurrence in the Haihe River, China. Environmental Science & Technology 44, 7220–7225. https://doi.org/10.1021/es100233w

Ng, L.K., Martin, I., Alfa, M., Mulvey, M., 2001. Multiplex PCR for the detection of tetracycline resistant genes. Molecular and Cellular Probes 15, 209–215. https://doi.org/10.1006/MCPR.2001.0363

Pei, R., Kim, S.C., Carlson, K.H., Pruden, A., 2006. Effect of River Landscape on the sediment concentrations of antibiotics and corresponding antibiotic resistance genes (ARG). Water Research 40, 2427–2435. https://doi.org/10.1016/j.watres.2006.04.017

Suzuki, M.T., Taylor, L.T., DeLong, E.F., 2000. Quantitative analysis of small-subunit rRNA genes in mixed microbial populations via 5’-nuclease assays. Applied and Environmental Microbiology 66, 4605–4614. https://doi.org/10.1128/AEM.66.11.4605-4614.2000
